# Can calmodulin bind to lipids of the cytosolic leaflet of plasma membranes?

**DOI:** 10.1098/rsob.240067

**Published:** 2024-09-18

**Authors:** Federica Scollo, Carmelo Tempra, Hüseyin Evci, Miguel Riopedre-Fernandez, Agnieszka Olżyńska, Matti Javanainen, Arunima Uday, Marek Cebecauer, Lukasz Cwiklik, Hector Martinez-Seara, Pavel Jungwirth, Piotr Jurkiewicz, Martin Hof

**Affiliations:** ^1^ J. Heyrovský Institute of Physical Chemistry of the Czech Academy of Sciences, Dolejškova 2155/3, 182 23 Prague 8, Czech Republic; ^2^ Institute of Organic Chemistry and Biochemistry of the Czech Academy of Sciences, Flemingovo nam. 2, 166 10 Prague 6, Czech Republic; ^3^ Department of Chemistry, Faculty of Science, University of South Bohemia in České Budějovice, 370 05 České Budějovice, Czech Republic; ^4^ Institute of Biotechnology, University of Helsinki, 00790 Helsinki, Finland

**Keywords:** calmodulin, lipid membrane, phosphatidylethanolamine, phosphatidylserine, calcium

## Abstract

Calmodulin (CaM) is a ubiquitous calcium-sensitive messenger in eukaryotic cells. It was previously shown that CaM possesses an affinity for diverse lipid moieties, including those found on CaM-binding proteins. These facts, together with our observation that CaM accumulates in membrane-rich protrusions of HeLa cells upon increased cytosolic calcium, motivated us to perform a systematic search for unmediated CaM interactions with model lipid membranes mimicking the cytosolic leaflet of plasma membranes. A range of experimental techniques and molecular dynamics simulations prove unambiguously that CaM interacts with lipid bilayers in the presence of calcium ions. The lipids phosphatidylserine (PS) and phosphatidylethanolamine (PE) hold the key to CaM–membrane interactions. Calcium induces an essential conformational rearrangement of CaM, but calcium binding to the headgroup of PS also neutralizes the membrane negative surface charge. More intriguingly, PE plays a dual role—it not only forms hydrogen bonds with CaM, but also destabilizes the lipid bilayer increasing the exposure of hydrophobic acyl chains to the interacting proteins. Our findings suggest that upon increased intracellular calcium concentration, CaM and the cytosolic leaflet of cellular membranes can be functionally connected.

## 1. Introduction

Calmodulin (CaM) is a multipotent regulator of diverse vital processes in cells. It converts changes in the intracellular calcium concentration to signalling events, the specificity of which is determined by its interaction with a variety of proteins. Up to four calcium ions bind to ‘EF hand’ sites (helix–loop–helix motifs) in the two globular domains [1] inducing conformational rearrangement of cytosolic apo-CaM to an open, ‘relaxed’ structure (holo-CaM). This is associated with the exposure of flexible hydrophobic pockets, which are involved in the binding of holo-CaM to a specific set of signalling proteins [2]. Typically, hydrogen bonds are formed between the two hydrophobic anchors of the amphipathic helices in target proteins and a hydrophobic pocket of holo-CaM. The large variability of binding motifs, however, indicates the extensive flexibility of CaM to accommodate diverse binding partners.

Since several targets of holo-CaM are membrane proteins [3], it is tempting to speculate that membranes play a role in CaM regulation or form a platform for the complex activity of this highly promiscuous protein. Earlier observations [4–6] indicated that increased expression of CaM in liver cells can cause a transient decrease in membrane fluidity upon an increase in intracellular calcium concentration. Whether CaM directly affects lipids in the membrane or the effect is mediated via CaM-binding proteins has not been addressed. Recent studies demonstrated that holo-CaM binds lipid moieties on several CaM-binding proteins [7–9]. For example, rising intracellular calcium concentration induces sequestration of the prenyl lipid moiety (farnesyl) of a small, highly oncogenic GTPase–KRas4b to the hydrophobic pocket of the C-terminal lobe in holo-CaM [10]. Moreover, Kovacs and co-workers demonstrated a direct interaction between CaM and a lipid, sphingosylophosphorylcholine (SPC), in the absence of other proteins [8]. SPC competed with CaM-target proteins indicating a regulatory role for this secondary messenger in CaM function [8]. A similar effect was observed for related lipids: sphingosine, galactosylsphingosine and glucosylsphingosine [11]. However, these results were obtained for either lipid monomers or micelles and do not address the interaction of CaM with membranes. Nevertheless, they suggest that CaM has the capacity to directly bind lipids, which opens the possibility that CaM actively uses cellular membranes for its function.

In this work, we address the question of whether CaM can bind to model lipid membranes mimicking the cytosolic leaflet of plasma membranes. Specifically, we studied the calcium-dependent interaction of CaM with lipid bilayers using a panel of membrane models and methods, i.e. confocal fluorescence microscopy, fluorescence correlation spectroscopy (FCS), surface plasmon resonance (SPR), generalized polarization (GP) and molecular dynamics (MD) simulations. All data demonstrate the ability of holo-CaM to associate with membranes containing both phosphatidylethanolamine (PE) and phosphatidylserine (PS) in the absence of other proteins.

## 2. Results and discussion

**Figure 1.**
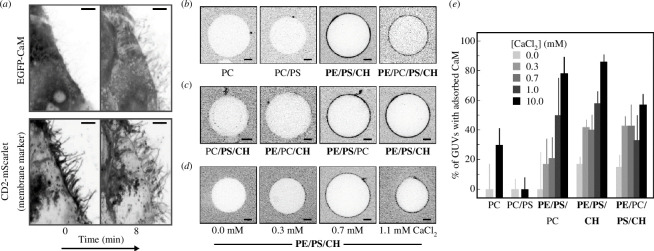
(*a*) Fluorescence confocal images of transfected HeLa cells expressing EGFP-CaM (upper images) and a membrane marker, CD2-mScarlet (lower images). Relocalization of EGFP-CaM to membrane protrusions is visible after ionomycin treatment. Scale bars, 5 μm. (*b,c*) Representative cross-sections of giant unilamellar vesicles (GUVs) of different lipid compositions (see table 1 for details) incubated with 30 nM CaM-R solution at 10 mM CaCl_2_. Scale bars, 5 μm. (*d*) Representative cross-sections of PE/PS/CH and 30 nM CaM-R at different CaCl_2_ concentrations: 0, 0.3, 0.7 and 1.1 mM. All the images were corrected for background intensity. Scale bars, 5 μm. *n* > 9 (≥3 different electroformations). (*e*) Percentage of GUVs showing CaM-R adsorption on their membranes for the studied lipid compositions at different CaCl_2_ concentrations.

In live-cell imaging experiments, we observed increased presence of CaM tagged with enhanced green fluorescence protein (EGFP-CaM) in membrane-rich protrusions of HeLa cells treated with ionomycin (figure 1*a* and electronic supplementary material, figure S2), which rapidly increases intracellular calcium concentration. In untreated cells, little to no signal of EGFP-CaM was detected in membrane protrusions. These results suggest that CaM can approach membranes upon Ca^2+^ intake. Is this solely due to CaM interaction with membrane proteins or can it directly associate with lipid bilayers? What would be the consequences of such association? To untangle this possibility and the role of specific lipids in a putative CaM–membrane interaction, we prepared several model membrane systems with the following compositions: phosphatidylcholine (PC), PC/PS (8:2), PE/PS/cholesterol (CH) (6:2:2), PE/PC/CH (6:2:2), PE/PS/PC (6:2:2), PC/PS/CH (6:2:2) and PE/PC/PS/CH (4:2:2:2) reported in table 1, where PC, PE, PS and CH stand for 1-palmitoyl-2-oleoyl-*sn*-glycero-3-phosphocholine, 1-palmitoyl-2-oleoyl-*sn*-glycero-3-phospho-ʟ-serine, 1-palmitoyl-2-oleoyl-*sn*-glycero-3-phosphoethanolamine and cholesterol, respectively (see electronic supplementary material, figure S1, for the chemical structures).

**Table 1.** Compositions of model lipid membranes used in this study.

	PC (mol%)	PS (mol%)	PE (mol%)	CH (mol%)
PC^a^ ^,b^	100	—	—	—
PE^b^	—	—	100	—
PC/PS^a,b^	80	20	—	—
PE/PS^b^	—	20	80	—
PE/PC^b^	20	—	80	—
PC/CH^b^	80	—	—	20
PE/CH^b^	—	—	80	20
PC/PS/CH^a^ ^,b^	60	20	—	20
PE/PS/CH^a^ ^,b^	—	20	60	20
PE/PC/CH^a,b^	20	—	60	20
PE/PS/PC^a^	20	20	60	—
PE/PC/PS/CH^a^	20	20	40	20

^a^ Used in the experiments.

^b^ Used in the simulations.

First, we studied the adsorption of rhodamine B labelled CaM (CaM-R) to giant unilamellar vesicles (GUVs) using confocal fluorescence microscopy.

Figure 1*b* demonstrates CaM-R adsorption to PE/PS/CH and PE/PC/PS/CH, but not to PC or PC/PS vesicles (GUVs). Since the adsorption was the most pronounced at PE/PS/CH membranes, we investigated which of its components promoted the effect by replacing one at a time with PC. From the resulting lipid compositions, PC/PS/CH, PE/PC/CH and PE/PS/PC, only the last one was effective (figure 1*c*). It is thus evident that both PE and PS were required for CaM-R adsorption, while CH was found to be dispensable. For those compositions in which CaM adsorption was noted at 10 mM Ca^2+^, the experiments were also repeated at lower, more physiological Ca^2+^ concentrations. The data are reported in figure 1*d,e* and indicate that CaM-R adsorbs to all the compositions containing both PE and PS lipids, with this interaction detectable already at 0.3 mM CaCl_2_.

**Figure 2.**
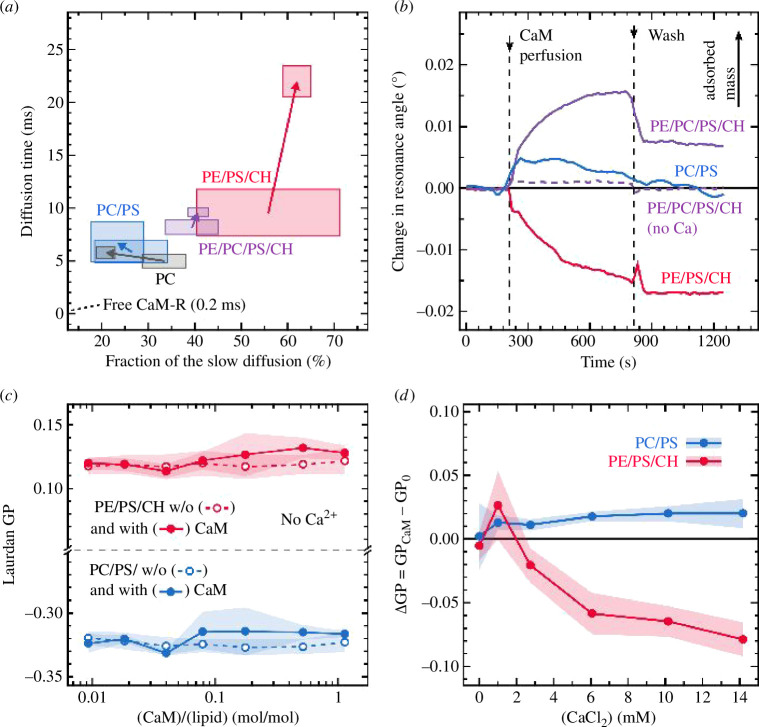
(*a*) Longer FCS diffusion time as a function of its fraction measured for 30 nM CaM-R diffusing at the surface of giant vesicles (GUVs) in the absence and in the presence of 10 mM CaCl_2_ (increasing CaCl_2_ concentration depicted by the arrows). Rectangles represent s.d., *n* > 5. (*b*) Change in the resonance angle measured using SPR for SPBs upon perfusion with 0.6 µM CaM solution. The solution contained 10 mM CaCl_2_ (solid lines) or no calcium (dashed line). Lipid composition of SPBs is given in table 1. Curves represent individual measurements. (*c*) Laurdan GP of LUVs as a function of CaM/lipid ratio in the absence of calcium. Open symbols and dashed lines represent control experiments without CaM. (*d*) ΔGP induced by CaM as a function of CaCl_2_ concentration for CaM/lipid ratio of 1.1. ΔGP is defined as GP for CaM-containing sample minus GP of a CaM-free sample. All measurements were performed at 37°C. Error bands represent s.e. (*n* = 3). Lipid compositions of all the model systems shown in this figure are given in table 1.

The observed CaM adsorption required rather long incubation times of >4 h, which contrasts with live-cell data. To approach earlier stages of the CaM–lipid membrane interactions, we took advantage of the sensitivity of FCS. Fluorescence fluctuations caused by CaM diffusion were measured in the bulk (solute) and at the surface of GUVs (see Methods in the electronic supplementary material for more details). The FCS data indicate that even in the absence of PE and Ca^2+^, there was a minute fraction of CaM at the surface of the GUV membrane in <30 min (figure 2*a*). However, the presence of PE, PS and CaCl_2_ increased the fraction of the CaM associated with membranes (figure 2*a*).

To avoid the impact of the fluorophore, we next used SPR—a label-free technique [12–15] enabling the determination of real-time protein adsorption to the surface of lipid membranes [16,17]. Supported phopholipid bilayers (SPBs) were created by vesicle deposition [18] on a gold two-channel SPR sensor covered with SiO_2_. Perfusion with 0.6 µM unlabelled CaM with 10 mM CaCl_2_ resulted in significant adsorption of CaM on PE/PC/PS/CH, which was stable even after subsequent washing with CaM-free buffer (figure 2*b*). The adsorption was detectable within a minute and increased during the period of the measurement (10 min). In the absence of calcium or PE, no CaM adsorption was detected. Increased PE content in this experiment resulted in membrane disintegration, as shown by the surface mass loss for PE/PS/CH in figure 2*b*. In analogy, while performing confocal microscopy, we observed GUV rupture after CaM-R addition to PE/PS/CH GUVs (≥40% of vesicles). This indicates that CaM-R can strongly perturb PE-containing membranes, up to the point where their integrity becomes compromised. Calcium-dependent binding of negatively charged proteins to PS-containing membranes is a well-known phenomenon [19,20]. However, how can one rationalize that PE is essential for holo-CaM binding to the lipid membrane? Calcium ions induce the conversion of apo-CaM into holo-CaM, exposing its hydrophobic pockets. As described before [7–10], a hydrocarbon tail of phospholipids can bind into these pockets. In membranes, however, the lipid tails are normally kept hidden in the bilayer core and not exposed to potential binding partners. Thus, CaM would need to surpass lipid–lipid interactions to access lipid acyl chains. We suggest that this process can be facilitated by PE—a cone-shaped lipid prone to destabilize the lamellar structure of a lipid membrane [21].

Confocal microscopy and SPR experiments indicated that holo-CaM can disintegrate PE/PS/CH membranes, which implies changes in the structural and dynamical properties of the lipid bilayer. To monitor such changes in membrane properties in the presence of CaM and calcium, we used GP of Laurdan [22]. This method probes the bilayer fluidity, more specifically the lipid mobility at the glycerol backbone level [23]. It is exceptionally sensitive to alterations in membrane physical properties [24], including those caused by proteins and peptides [25–27]. In the absence of calcium, no effects of CaM on PC/PS and PE/PS/CH large unilamellar vesicles (LUVs) were detected (figure 2*c*). Figure 2*d* reports on the direct effect of CaM on the GP, removing the contribution of the change in the GP caused by calcium in the absence of CaM (ΔGP) as a function of calcium concentration. In the presence of increasing calcium concentration, CaM caused a significant decrease in ΔGP of PE/PS/CH LUVs at 3 mM CaCl_2_ and above. Noteworthy, a decrease in membrane rigidity represented by a decrease in the GP value upon the binding of any other protein has not been reported in earlier studies. We suggest that membrane destabilization caused by CaM–PE interaction is the reason for the lowering of Laurdan ΔGP observed here for PE/PS/CH in the presence of Ca^2+^ (figure 2*d*). In the case of PC/PS membranes, a small, but statistically significant elevation of ΔGP can be observed already at 1 mM CaCl_2_, and it does not further increase at larger Ca^2+^ concentrations. In analogy to our previous work [25,27–30], this mild rigidification of the lipid bilayer might suggest a peripheral association of CaM with the lipid bilayer. For CaM, we discovered the strongest calcium-dependent alteration of the headgroup mobility for the PE-containing bilayers but in a unique direction (figure 2*d*). Evidently, CaM perturbs the cohesive interactions between the lipid molecules, which is consistent with the disruptive interactions observed between CaM and PE/PS/CH membranes.

**Figure 3.**
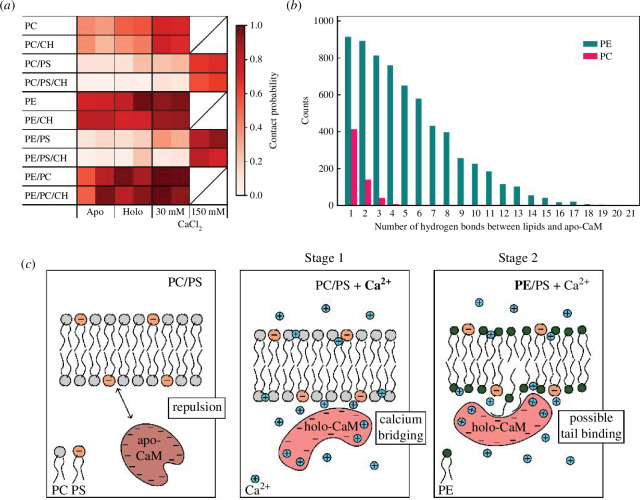
(*a*) Probability of contact between CaM and lipid membranes. Rows represent different lipid compositions (see table 1 for details). Columns represent different CaM forms and CaCl_2_ concentrations. Each cell contains two different replicates of the simulations (each 5 µs long). Details of the simulations are provided in electronic supplementary material, table S1. (*b*) Histograms of hydrogen bonds between apo-CaM and membranes for PC and PE membranes. (*c*) Molecular sketch illustrating our hypothesis of two-stage model of CaM binding to lipid bilayer. In the absence of calcium ions, apo-CaM is electrostatically repelled from the negatively charged surface of PC/PS membrane (left sketch). Calcium causes formation of holo-CaM exposing its hydrophobic binding pocket. Calcium ions also adsorb to negatively charged surfaces of holo-CaM and PC/PS membrane bridging them (stage 1, middle sketch). This process can cause transient association of CaM with the membrane. PE forms hydrogen bonds with holo-CaM, which further attracts the protein to the membrane. PE also destabilizes the membrane, increasing the probability of exposure of hydrophobic lipid tails and their binding to the hydrophobic pocket of CaM (stage 2, right sketch). This could lead to membrane rearrangement and stable CaM adsorption or to destruction of the membrane.

The different experimental approaches described above reveal three components that favour the adsorption of CaM to lipid bilayers: PE and PS lipids as well as calcium ions. To better understand the molecular nature of the initial step in CaM adsorption and to get insights into the role of these three components, we ran atomistic multi-microsecond MD simulations of CaM (Protein Data Bank identification code 1CLL that transitioned to a more compact form) at membranes with the lipid compositions used in the experiments and the following additional ones: PE, PE/PS (8:2), PE/PC (8:2), PC/CH (8:2) and PE/CH (8:2) (see table 1, Experimental Procedure and electronic supplementary material, table S1, for a detailed description of the systems). Briefly, CaM was initially placed in the aqueous phase, distant from the studied membranes, each of which had different compositions and each contained a total of 200 lipids. Then, CaM was allowed to spontaneously interact with the membrane during a 5-µs-long simulation (two repeats for each system). To model the interactions among charged species, we used the implicitly polarizable simulation models that have captured the calcium-bridged bridging of the PKC*α*-C2 domain onto PS lipids in our earlier work (see electronic supplementary material for details) [31]. The contact probability of membranes with apo- and holo-CaM (four calcium ions bound to the calcium-binding sites) at different CaCl_2_ concentrations was calculated from the simulations. The data are represented in figure 3*a*.

In agreement with the experiments, MD simulations demonstrate that exchanging PE with PC in tested membranes lowered the probability of their contact with CaM (apo-CaM and holo-CaM at any calcium concentration). This difference can be explained at the molecular level by the higher capability of PE to form hydrogen bonds with the protein (figure 3*b*). Interestingly, the contact probabilities in the absence of calcium ions in solution drop dramatically once PS is added to the lipid mixtures, likely because of the net negative charge of PS, which repels CaM, a highly negatively charged protein, and prevents its contacts with the membrane. However, when Ca^2+^ concentration is high, the contact probability of CaM with PS-containing membranes significantly increases, i.e. to about 80% at 150 mM of calcium (figure 3*a*). Indeed, the contact probability of CaM with membranes increases with calcium concentration to a different extent but consistently for all membrane compositions, indicating that calcium ions are key for the protein to approach the membrane (figure 3*a*). While this concentration of calcium seems high compared to the experiments, the simulations actually contain a modest total number of ions, and in experiments, its local concentration in the vicinity of charged membranes might be similarly elevated. We observed similar calcium dependence for CaM presence in membrane-rich protrusions but cannot exclude protein mediators in live-cell experiments (figure 1*a*). The presence of cholesterol appears dispensable for the protein–membrane binding for all the simulated compositions, confirming the experimental results in figures 1 and 2. Additional simulations show that for systems containing PE and Ca^2+^, binding modes with more EF loops bound at the same time occur (electronic supplementary material, figure S6). The data suggest that the probability that CaM lies down on the membrane surface is increased by the presence of PE.

Based on our results and previous studies [8,9], we propose a two-stage model of CaM–membrane interactions, illustrated in figure 3*c*. As demonstrated before, the adsorption of Ca^2+^ to PC/PS membrane can neutralize its negative charge or even turn it positive (in case of overbinding [32]). Our MD simulations indicate that Ca^2+^ bridging might be the driving force for initial CaM interactions with PS-containing membranes (stage 1, figure 3*c*, middle panel). This process can lead to a transient association of CaM with the membrane, which we consider the first necessary stage for the binding to occur. PE likely plays a dual role: (i) its ability to form hydrogen bonds with the holo-CaM can accelerate CaM attraction to the membranes and (ii) PE, with its preference for non-lamellar structures, destabilizes lipid bilayers, which can lead to the exposure of lipid tails to the hydrophobic pocket of holo-CaM (stage 2, figure 3*c*). Such events may lead to a more stable interaction of CaM with membranes.

The explained model was additionally strengthened by zeta potential and dynamic light scattering (DLS) measurements reported in electronic supplementary material, figures S4 and S5. The data show that adding CaM to vesicles containing PE and PS in the presence of calcium decreases the zeta potential, further proving the calcium-dependent binding of CaM to membranes containing those lipids. Moreover, DLS data indicate that CaM is directly involved in the formation of liposomal aggregates (see electronic supplementary material, figures S4 and S5).

What makes the interaction of CaM with membranes mimicking the cytosolic leaflet of cellular membranes unique? Ca^2+^ dependence of protein association with membranes is a well-described phenomenon (e.g. for the C2 domain of phospholipase A2 [33]). Anionic phospholipids regulate the function of numerous membrane-associated signalling molecules (e.g. Akt [34]). However, none of these Ca^2+^- and/or lipid-dependent proteins exhibits such an interplay between PE and PS.

In general, the direct regulation of proteins by PE is less well understood than that by PS. It has been shown that PE facilitates the interaction of proteins bearing a hydrophobic surface with membranes [35]. Moreover, PE has an indirect positive effect on the interactions of proteins with negatively charged membranes [36,37]. This is especially pronounced at lower content of PS. In the case of coagulation factors, PS and PE functioned in synergy: PS was sufficient for a protein binding to membranes, but the presence of PE reduced *K*_D_ values of this interaction [36,37]. A similar positive effect of PE was observed for protein binding to diacylglycerol-containing membranes (e.g. protein kinase C [36]). PE and PS are metabolically closely related, which can also be reflected in their functions [38]. We believe that in the case of holo-CaM binding to membranes, PE and PS might as well work synergistically. Lanthionine-containing peptide antibiotics, duramycin and cinnamycin, bind to lipid bilayers in a strictly PE-dependent manner [39]. At high PE concentrations, these peptides destabilize membranes and cause cell death [40]. This is indeed similar to our observation that holo-CaM can destabilize PE-containing membranes at higher calcium concentrations. Since the formation of membrane pores, as shown for cinnamycin, is highly improbable [40], we hypothesize that holo-CaM may bind to the phospholipids released by the PE-containing membranes or to lipid acyl chains exposed at the surface.

What supports our view about CaM–acyl chain interactions? The prenyl moiety of KRas4b binds to the hydrophobic groove of holo-CaM, regulating its function [41]. Similar processes were described for holo-CaM interacting with prenylated RalA [7]. Moreover, holo-CaM binds the CAP23/NAP22 protein by accommodating its myristoyl moiety in its hydrophobic pockets [42]. This indicates that alkyls can facilitate holo-CaM binding as efficiently as prenyls. Basic residues in the proximity of the prenylation (myristoylation site) facilitate holo-CaM attraction to the target lipidated proteins and stabilize the bimolecular complex. Such a scenario resembles our observation that, in the presence of Ca^2+^, PS attracts CaM to the membrane, but PE is required for membrane destabilization and acyl chain exposure for a more stable binding of holo-CaM (figure 3*c*).

## 3. Conclusion

Our study demonstrates that, under certain conditions, CaM, which is normally a hydrophilic, highly negatively charged and well-soluble protein, can interact with model lipid membranes. It revealed the specific involvement of PS and PE lipids, which are major components of the cytosolic leaflet of the plasma membrane. Based on our results and the existing literature, we propose a two-stage model of CaM interaction with membranes: (i) Ca^2+^ adsorption to PS headgroups facilitates transient interactions of holo-CaM with a bilayer, which is supported by the formation of hydrogen bonds between holo-CaM and PE; (ii) PE, by reducing cohesive lipid interactions and potentially increasing probability of acyl chain exposure, further promotes and stabilizes CaM adsorption to membranes (figure 3*c*). The latter can lead to destabilization or even disintegration of model membranes. While the role of PS follows a typical pattern found in calcium-mediated protein–membrane interactions, PE acts in a unique fashion. Overall, our study provides a mechanistic insight into the intricate interplay between CaM and membranes, suggesting a possible regulatory role of membrane lipids in the complex activity of CaM.

## Data Availability

The experimental data are available at the Zenodo repository: doi.org/10.5281/zenodo.10843995 [43], doi.org/10.5281/zenodo.12795856 [44]. The computational data are available at the Zenodo repository: doi.org/10.5281/zenodo.10727235 [45], doi.org/10.5281/zenodo.10727430 [46], doi.org/10.5281/zenodo.10727572 [47]. Supplementary material is available online [48].
